# Project of Neutron Beta-Decay A-Asymmetry Measurement With Relative Accuracy of (1–2)×10^−3^

**DOI:** 10.6028/jres.110.057

**Published:** 2005-08-01

**Authors:** A. Serebrov, Yu. Rudnev, A. Murashkin, O. Zherebtsov, A. Kharitonov, V. Korolev, T. Morozov, A. Fomin, V. Pusenkov, A. Schebetov, V. Varlamov

**Affiliations:** PNPI, St. Petersburg Nuclear Physics Institute, 188300, Gatchina, Russia

**Keywords:** beta-decay, cold neutrons, polarization

## Abstract

We are going to use a polarized cold neutron beam and an axial magnetic field in the shape of a bottle formed by a superconducting magnetic system. Such a configuration of magnetic fields allows us to extract the decay electrons inside a well-defined solid angle with high accuracy. An electrostatic cylinder with a potential of 25 kV defines the detected region of neutron decays. The protons, which come from this region will be accelerated and registered by a proton detector. The use of coincidences between electron and proton signals will allow us to considerably suppress the background. The final accuracy of the *A*-asymmetry will be determined by the uncertainty of the neutron beam polarization measurement which is at the level of (1–2) × 10^−3^, as shown in previous studies.

## 1. Introduction

An improved measurement of the neutron *β*-decay *A*-asymmetry is extremely important to test the Standard Model of interaction of elementary particles. In the framework of the Standard Model of *V*-*A*-weak interactions the probability of neutron *β*-decay is written in the following form:

$$W\left(E_{e,}p_{e},p_{\tilde{v}}\right)=f\left(E_{e}\right)\left[1+a\frac{v}{c}\cos\left(p_{e},p_{\tilde{v}}\right)+A\frac{v}{c}P\cos\left(\sigma ,p_{\tilde{v}}\right)+BP\cos\left(\sigma ,p_{\tilde{v}}\right)\right],$$ (1)

where *W*(*E*_e_, ***p***_e_, $p_{\tilde{v}}$) is the probability of neutron *β*-decay; *E*_e_, ***p***_e_ are the energy and momentum of the electron; $p_{\tilde{v}}$ is the momentum of the antineutrino; *v* is the velocity of the electron; *a*, *A*, *B* are the correlation coefficients; ***σ*** is the neutron spin; *P* is the neutron beam polarization.

The coefficient *A* is the most sensitive to the *λ*-value, which is the ratio of fundamental coupling constants $\left(\lambda =G_{A}/G_{V}\right).A_{0}=-2\lambda \left(\lambda +1\right)/\left(1+3\lambda^{2}\right);\frac{\Delta \lambda }{\lambda }=0.25\frac{\Delta A}{A}$. Using the precise measurement of the neutron lifetime and *A*-asymmetry it is possible to determine the *V_ud_* element of the CKM matrix. The analysis of unitarity of CKM from the work [1] and a new result for the neutron lifetime [2] require improved accuracy for the *A*-asymmetry measurement. The most precise experiment [3] gives *A*_0_ = −0.1189(7) and *λ* = −1.2739(19). The required level of accuracy has to be 2 × 10^−3^ or better to reach the comparable accuracy of *V_ud_* determination from neutron *β*-decay and from high quark generation decay.

We are going to reach the necessary level of accuracy using a new experimental scheme for the *A*-asymmetry measurement.

## 2. The Scheme of the Proposed Experiment

**Fig. 1:**
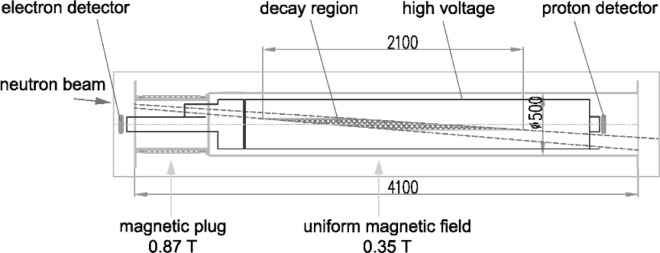
Scheme of correlation spectrometer.

The scheme of the correlation spectrometer is shown in Fig. 1. The correlation spectrometer is itself a solenoid with a uniform magnetic field and a magnetic plug at one of its ends. Such a magnetic system acts as a magnetic collimator for decay electrons. Electrons whose momentum with respect to the axis are inside the solid angle *θ_C_* can go through the magnetic plug field and reach the electron detector. Electrons outside this solid angle will be reflected by the magnetic field of the plug. The angle *θ_C_* is defined by the ratio of the magnetic field in the uniform part and in the magnetic plug, and it is not dependent on the energy of electron

$$\sin^{2}\theta_{C}=H_{O}/H_{m},$$ (2)

where *H*_O_ is the magnetic field value in the region of neutron decay (uniform part) and *H*_m_ is the magnetic field value in the magnetic plug.

**Fig. 2:**
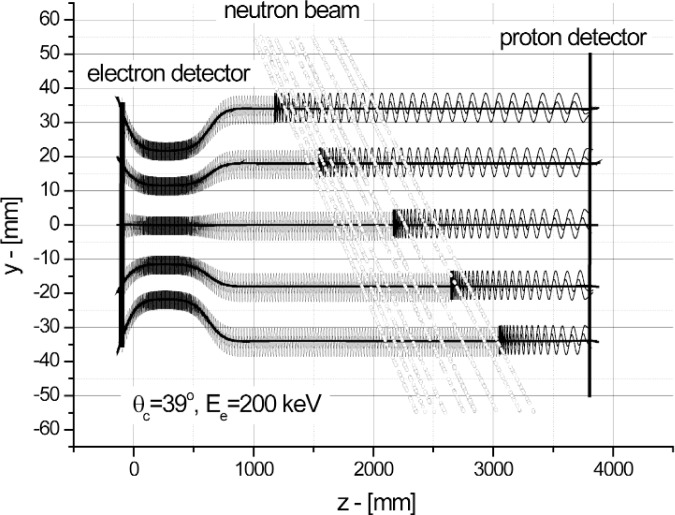
Magnetic field and trajectories of electrons and protons. (Scales for y and z directions are different by a factor of 30).

The long solenoid *L* = 3320 mm produces a magnetic field *H*_O_ = 0.35 T. The short solenoid produces a magnetic field *H*_m_ = 0.87 T. For this configuration of magnetic fields *θ*_C_ is equal to 39 °. The electron and proton detectors are installed on the axis of the magnetic system. The region of detected *β*-decay events is defined by the intersection of the neutron beam with the magnetic field lines that go through the electron detector. This region is crosshatched in Fig. 1. The size of the proton detector has to be large enough to collect all protons related to the detected electron. Fig. 2 shows the magnetic field lines and trajectories of electrons and protons around the lines. The condition of 100 % collection of protons is very important as the average cosines for the *a* and *B* correlation coefficients will be equal to zero in this case. Therefore we can measure the *A* correlation coefficient without any correction for admixtures of other correlation coefficients. The signals from the electron and proton detectors have to be detected in coincidence to fulfill this condition. Additionally the use of a coincidence regime of registration of *β*-decay events will allow us to considerably suppress the background, and to separate effect and background by means of the delayed coincidence method.

To detect the protons which have very low initial energy, the decay region is placed at high voltage (25 kV). When protons leave the high voltage region they are accelerated up to the energy of 25 keV and they can be registered by the proton detector. The electrostatic system of the correlation spectrometer consists of a multi-section cylinder with very thin nets at the ends. The sections of the electrostatic system are under different potentials from 21 kV up to 26 kV. Due to the gradient of high voltage potential an electric field appears inside the cylinder that is aligned along the axis of the spectrometer. All protons will be accelerated in the direction of the proton detector independently of their initial momentum. This scheme of the electrostatic system allows us to collect all the protons and to obtain zero average cosines of *a* and *B* correlations. The protons can be collected during a time of less than 10 µs. This helps to reduce the background of random coincidences.

The main task of the experimental procedure is the measurement of the experimental asymmetry which is defined in the following way:

$$X=\frac{N↑-N↓}{N↑+N↓}=A\frac{v}{c}P\bar{\cos}\left(\sigma_{n},p_{e}\right)$$ (3)

where *N*↑ and *N*↓ are the counts of coincidences for different directions of neutron beam polarization.

Equation (3) indicates that a relative accuracy of *A*-asymmetry measurements of 10^−3^ can be reached when all values: the average cosine of the *A* correlation coefficient $\bar{\cos}\left(\sigma_{n}p_{e}\right)$, the neutron beam polarization *P*, the electron velocity $\frac{v}{c}$ and the experimental asymmetry *X* are measured or determined with a relative uncertainty better than 10^−3^.

## 3. Determination of Average Cosine of *A* Correlation Coefficient $\bar{\cos}\left(\sigma_{n,}p_{e}\right)$

The uncertainty of determination of the average cosine $\bar{\cos}\left(\sigma_{n}p_{e}\right)$ depends on the homogeneity of the magnetic fields in the decay region and in the region of the magnetic plug. For $\frac{H_{0}}{H_{m}}=0.4$ and $\frac{\Delta H}{H}\approx 2\times 10^{-3}$ we have: $\frac{d\bar{\cos}\left(\sigma_{n},p_{e}\right)}{\bar{\cos}\left(\sigma_{\text{n},}p_{e}\right)}=1.3\times 10^{-4}$. Thus the average cosine of the *A* correlation coefficient can be determined with very high accuracy. It can be attained by the magnetic collimation method, which is independent of the position of neutron *β*-decay.

## 4. Detectors of Electrons and Protons—Determination of *v/c* Value

The electron detector will have a size of 55 × 160 mm^2^. We plan to use a Si(Li)-detector for registration of electrons. This detector will be divided into 16 individual sections. Previously we performed a test of a Si(Li)-detector with a diameter of 70 mm and a thickness of 3.5 mm to measure the backscattering effect of electrons from Si. A magnetic *β*-spectrometer was used for this purpose. Thanks to these measurements we can distinguish the tail of the 976 keV line with total area 12 %. In our measurement with the correlation spectrometer the effect of electron backscattering will be even more suppressed because the backscattered electrons will be reflected again from the magnetic plug with probability about 80 %. Those backscattered electrons that do go through the magnetic plug, will be registered by the proton detector. This fraction will be about 2.5 % only. We can reject these events due to a signal of prompt coincidence from both detectors. Therefore the energy resolution of the electron detector will be high enough. The energy calibration of this detector can be done by means of a set of conversion sources.

The proton detector will have a size of 75 × 200 mm^2^, i.e., 20 mm broader and 40 mm higher than the size of the electron detector. This is done to insure 100 % collection of protons. The proton detector will have 16 sections as does the electron detector. This will help us to suppress the background of random coincidences and is necessary in any case to reduce the capacitance of the detector. We are going to use surface barrier detectors made from pure silicon. As a possible alternative to this we are considering the use of microchannel plates.

## 5. Precise Collimation of Neutron Beam

The task of precisely collimating the neutron beam is very important for our experimental scheme because the neutron beam passes close to the detectors.

The most appropriate collimator material is ^6^Li, which practically does not produce any *γ* rays after neutron capture. Because of its chemical activity, ^6^Li is used in form of ^6^LiF with some ingredients added to reduce the brittleness of LiF-ceramics. The small angle scattering effect from this material was studied to obtain initial data for the design of precise collimators.

The experiment was carried out at the PNPI reflectometer of the WWR-M reactor. Using the data of the small angle scattering process that was obtained, a multi-diaphragm collimator has been designed. The distribution of neutron intensity at a distance of 4 m from the collimator was calculated. It was shown that the suppression factor of intensity reaches eight orders of magnitude at a distance of 2 cm to 3 cm from the edge of the beam. This result must be checked experimentally to be sure that satisfactory background conditions can be reached.

## 6. The Measurements of Neutron Beam Polarization With an Accuracy (1–2) × 10^−3^

The possibility of precise measurements of neutron beam polarization has been studied in our detailed experiments [4, 5]. The scheme of the proposed method consists of two analyzers and two double flippers.

The comparison of the two analyzers and two flippers method with the method of ^3^He filters was done in our work [5]. It was shown that the correction for the supermirror method is 0.152(18) %. Thus, usage of the mentioned methods of neutron beam polarization determination can give measurement accuracies of 10^−3^ and better.

## 7. Statistical Accuracy of the Experiment and the Random Coincidence Background

The statistical accuracy of the experimental asymme-1 try Δ*X* is defined by $\frac{1}{\sqrt{N}}$, therefore 10^8^ events have to be registered to obtain the accuracy Δ*X* ≈ 10^−4^.

The neutron flux density (*Φ*) at the exit of the polarized cold neutron beam of PSI fundamental facilities “Funspin” is about 2 × 10^8^ cm^−2^s^−1^. The neutron flux density after the collimator will be one order of magnitude less (*Φ*_C_ = 0.15 × 10^8^ cm^−2^s^−1^). We expect that the counting rate of neutron *β*-decay event (in coincidence) will be about 110 s^−1^. Therefore 10^8^ events will be collected during two weeks. In this estimation it was assumed that the background of random coincidences is considerably less than the signal.

The estimation of the background of the random coincidence can be done using the background counting rate of the electron and proton detectors in similar experiments [6, 7]. The background of the random coincidences with a resolution time of 10 µs should be about (2 to 20) s^−1^.

In order to obtain an exact answer concerning the background conditions, the test experiment with the collimated neutron beam and the electron and proton detectors is required.

## 8. Conclusion

The above-mentioned considerations show that the proposed experiment could reach an accuracy of *A*-asymmetry measurement at the level of (1–2) × 10^−3^. As a first step of the realization of the experiment, test measurements of background should be carried out.

Additionally, it should be mentioned that this spectrometer allows one to measure the *B* and *a* correlation coefficients as well. To do that, it is necessary to switch off the accelerating electric field in the spectrometer and to measure the longitudinal proton momentum by means of the time-of-flight method. Another way is to use the electrostatic spectrometer in front of the proton detector.
